# Evidence-based recommendations for the use of continuous glucose monitoring in type 2 diabetes: the Italian guidelines

**DOI:** 10.1007/s00592-026-02693-6

**Published:** 2026-05-28

**Authors:** A. Giaccari, G. Gliozzo, G. Ciccarelli, G. Di Giuseppe, C. Castellano, S. Cum, L. Delle Monache, M. Gallo, M. Lastretti, G. Medea, M. Monesi, R. Napoli, B. Pintaudi, E. Succurro, G. Turchetti

**Affiliations:** 1 Center for Endocrine and Metabolic Diseases, Fondazione Policlinico Universitario A. Gemelli IRCCS and Università Cattolica del Sacro Cuore, Rome, Italy; 2 Azienda USL of Modena, Sassuolo Hospital, Sassuolo, Italy; 3 Diabetes and Diabetic Foot Care Unit, ASUGI, Monfalcone, Italy; 4 National Board Member of FAND (Italian Association for the Rights of Diabetic People), Roma, Italy; 5 Department of Endocrinology and Metabolic Diseases, AO SS. Antonio e Biagio e Cesare Arrigo, Alessandria, Italy; 6 Order of Psychologists of Lazio, Rome, Italy; 7 Italian Society of General Medicine (SIMG), Florence, Italy; 8 Territorial Diabetology Unit, AUSL Ferrara, Ferrara, Italy; 9 Department of Translational Medical Sciences, University of Naples Federico II, Naples, Italy; 10 Diabetes Unit, Niguarda Cà Granda Hospital, Milan, Italy; 11 Department of Medical and Surgical Sciences, Magna Graecia University, Catanzaro, Italy; 12 Institute of Management, Scuola Superiore Sant’Anna, Pisa, Italy; 13 Patient Advocacy Lab, ALTEMS – Università Cattolica del Sacro Cuore, Rome, Italy

**Keywords:** CGM, Type 2 Diabetes, Metanalysis, PICO, GRADE, Guidelines

## Abstract

**Background and aims:**

Although continuous glucose monitoring (CGM) devices are now standard of care among Type 1 diabetes patients, they are still relatively underutilized in Type 2 diabetes (T2D), particularly in those patients not treated with insulin. Widespread adoption continues to be hindered by a combination of factors. Chief among these is the scarcity of long-term, large-scale clinical trials demonstrating the benefits of the use of CGM in T2D. This meta-analysis aimed to address this gap by comparing CGM with self-blood glucose monitoring (SBMG), with primary outcomes of HbA1c and time in range (TIR) in insulin-treated and non-insulin-treated TD2 patients.

**Methods and results:**

Following the stringent rules mandated by our National Health Service (which requires a panel composed of all stakeholders involved in diabetes treatment, and includes PICO, GRADE, AGREE, and meta-analyses), we performed a systematic review of RCTs that enrolled two groups of individuals with T2D, those treated with insulin (including basal and basal-bolus regimens), and those receiving treatments other than insulin. All included trials compared CGM with structured blood glucose monitoring (SBGM) with glycated hemoglobin (HbA1c) as the main endpoint. Based on the strength and consistency of the evidence, the panel issued a strong recommendation in favor of CGM for individuals with T2D treated with insulin (including those on basal insulin alone) and for individuals with T2D not treated with insulin, particularly for those with glycated hemoglobin levels ≥ 7%. From a pharmacoeconomic perspective, outcomes were positive in both patient groups.

**Conclusion:**

CGM represents a clinically effective and cost-efficient approach to optimizing glycemic control in T2D, becoming mandatory among individuals on insulin therapy. Our findings support a shift in clinical practice toward the more widespread use of CGM in T2D, with regulatory frameworks and reimbursement policies needing to adapt accordingly.

**Supplementary Information:**

The online version contains supplementary material available at 10.1007/s00592-026-02693-6.

## Introduction

Diabetes is a major and rapidly growing global health issue, which is accompanied by a range of micro and macrovascular comorbidities that significantly impact mortality and healthcare costs. Several studies have shown that containing risk factors within target ranges greatly improves outcomes in terms of these complications. In 1998, the United Kingdom Prospective Diabetes Study (UKPDS) was the first to show that intensive glucose control in patients newly diagnosed with type 2 diabetes (T2D) reduced the risk of microvascular complications [1] with these results continuing to be borne out in the subsequent follow-ups [2]. Since then, several observational and cross-sectional studies have shown associations between lower glycated hemoglobin (HbA1c) levels and reduced risk of complications, [3–5] even though the ADVANCE trial, [6] which was specifically designed to examine the effects of HbA1c reduction on macrovascular and microvascular outcomes or mortality, demonstrated only microvascular benefits with no robust evidence for macrovascular benefits, and, additionally, an increase in mortality.

In specific trials, several drugs have been shown to significantly reduce major adverse cardiovascular events (MACE), leading the 2018 EASD-ADA Consensus Guidelines to recommend them as priority treatments for high risk patients and those requiring secondary prevention [7]. However, the cardioprotective effects should not overshadow the importance of achieving target HbA1c levels, which remains a fundamental goal for all patients with type 2 diabetes. In other words, while these drugs confer significant cardiovascular benefits, optimal glycemic control remains essential to achieve prevention of both microvascular and macrovascular complications. Randomized controlled trials (RCTs) of newer glucose-lowering drugs, such as GLP-1 receptor agonists (e.g., LEADER) [8], and SGLT-2 inhibitors (e.g., EMPA-REG OUTCOME) [9], have shown a reduction in HbA1c levels and in major cardiovascular events in type 2 diabetes. Although these studies were designed to assess cardiovascular outcomes, intervention groups consistently achieved lower HbA1c. This improved glycemic control may partly explain the observed benefits, but long-term efficacy has yet to be proven.

While HbA1c is the gold standard for evaluating the risk of complications in diabetes, to date recommended HbA1c targets vary. The American Diabetes Association (ADA) 2026 Standards of Care [10] recommend that most T2DM patients maintain levels of glycated hemoglobin < 7% (53 mmol/mol) and sets a more ambitious target of HbA1c ≤ 6.5% (48 mmol/mol) for newly diagnosed adults, without comorbidities and low hypoglycemia risk. The Italian guidelines, elaborated on the basis of a very strict methodology, provide similar recommendations to the AACE, and suggest values of ≤ 6.5% (48 mmol/mol) in patients on low hypoglycemia risk treatments, i.e., without insulin or sulfonylureas. [11]

Global population estimates show that an ample proportion of individuals with diabetes do not meet glycemic targets, the median proportion achieving an HbA1c < 8% being ~ 68%, [12] although target attainment was higher in studies conducted in Europe and North America than in the rest of the world [13]. Given the importance of achieving and maintaining target values, any strategy or intervention that supports this goal should be incorporated into the therapeutic approach for the management of type 2 diabetes.

Continuous glucose monitoring (CGM) has revolutionized diabetes management, particularly in individuals with type 1 diabetes, where it is now considered the standard of care. [14, 15] Beyond adapting insulin treatment to glucose levels, CGM allows patients to observe how food, medications, stress, physical activity, sleep patterns, illness, hormonal fluctuations, and alcohol intake affect their glycemic levels [16]. In a systematic metanalysis, Richardson et al. showed that by allowing users to see the almost immediate effects of their actions, CGM served as a biofeedback loop leading to changes in behavior in subjects with, and even in those without, diabetes. Improved patient awareness led to improved self-management in terms of choice of foods, timing/size of meals, increased activity and management of medications, translating into improved HbA1c levels and Time In Range (TIR). The latter, usually defined as the percentage time spent with blood glucose concentrations between 70 and 180 mg/dl has emerged as a key metric in diabetes management. Notably, TIR is increasingly used as a primary endpoint in many trials, even in those involving patients with T2D, [17] emerging as a counterpart to HbA1c in clinical importance. Notwithstanding, CGM remains relatively underutilized in subjects with type 2 diabetes, particularly among those not in treatment with insulin. Widespread adoption continues to be hindered by multiple factors, foremost among them the lack of long-term, large-scale clinical trials demonstrating the benefits of CGM in T2D. Although CGM has been shown to reduce hypoglycemic episodes, and increased hypoglycemia is associated with higher mortality, these conclusions are derived from associative data rather than evidence from randomized trials. Furthermore, there are economic concerns, since the cost of CGM devices can be expensive for both health systems and individual patients. In Italy, the complex landscape of national regulatory pathways creates additional economic concerns for marketing of the devices, as decisions about devices and their reimbursement are decentralized to the regions. Given Italy’s 20 + regions, there is great variability as to how CGM is reimbursed, which patient groups qualify and what devices/systems are covered. Moreover, due to the rapidity of technological innovation in the field, leading to high product turnover, there are no long-term trials to evaluate the use of CGM. This high turnover rate also discourages investment from healthcare institutions and creates uncertainty among clinicians and patients as to which devices to adopt.

The 2025 Standards of Medical Care in Diabetes, issued by the American Diabetes Association (ADA), introduced a strong recommendation for CGM use in individuals with T2D treated with insulin (Recommendation 7.15), and a weaker, conditional statement supporting its use in non-insulin-treated T2D patients (Recommendation 7.16) [18]. Both were confirmed (in recommendation 7.15) in the 2026 update [19]. These recommendations were based on a systematic review of available clinical trials and real-world evidence, interpreted by an expert panel, a well-established methodology in the formulation of guidelines. Nevertheless, the evidence underlying the conditional recommendation for non-insulin-treated T2D patients (Recommendation 7.16) remains limited, and the present meta-analysis aims to quantify the effect of CGM on key glycemic outcomes in this population, examining only randomized clinical trials.

The Italian guidelines are based on a rigorous evaluation of the evidence. In Italy, the development and updating of clinical guidelines is a centralized process coordinated by the Italian National Institute of Health (Istituto Superiore di Sanità—ISS). Guidelines must adhere to stringent procedural frameworks including the use of PICO (Population, Intervention, Comparator, Outcome) formulations, evidence grading using the GRADE methodology, and a formal evaluation of cost-effectiveness. The process also mandates the inclusion of a multidisciplinary panel of stakeholders, including not only healthcare professionals but also patient representatives, nurses, dietitians, and economists. In 2022 the Italian Diabetes Society (SID) and the Association of Diabetologists (AMD) created a panel to develop guidelines on the basis of the above methodology. Following the publication of Italian national guidelines for the treatment of T2D in 2022, and an initial update in 2023 [11], the guideline panel convened once again in 2024 (prior to the publication of the 2025 ADA guidelines) to reassess the role of CGM in T2D in the light of emerging evidence. This article summarizes the methods and findings of this evaluation and presents the rationale and outcomes of the new recommendations adopted by the national guideline panel.

## Methods

### Guideline development process

The panel, appointed by AMD and SID to update the guidelines consisted of thirteen members, including six diabetologists (one of whom served as the coordinator), a general practitioner, a nurse, a dietitian, a psychologist, a pharmacoeconomist, and a representative of patient advocacy organizations. The panel followed procedures compliant with the AGREE II framework, which emphasizes transparency, rigor, stakeholder engagement, and editorial independence.

The first step in the process was the identification and prioritization of key clinical questions, which were then translated into PICO format. After extensive discussion, the panel decided to assess CGM use in two distinct populations: (1) individuals with T2D treated with insulin (including both basal and basal-bolus regimens), and (2) individuals with T2D not treated with insulin. Comparisons were made against standard self-monitoring of blood glucose (SMBG), which remains the conventional method in most clinical settings.

The panel designated the following outcomes for evaluation:

*Critical outcomes*: Reduction in glycated hemoglobin (HbA1c); increase in Time in Range (TIR; 70–180 mg/dL).

*Non-critical outcomes*: Reduction in Time Below Range (TBR; < 70 mg/dL).

### Literature review and evidence synthesis

A comprehensive systematic literature search was conducted by an independent evidence review team. The databases searched included PubMed (see supplementary PubMed search definitions), the Cochrane Central Register of Controlled Trials, EMBASE, and ClinicalTrials.gov. Importantly, only randomized controlled trials (RCTs) that reported at least one of the prespecified outcomes and compared CGM with SMBG in the target populations were included.

We initially identified a total of 937 articles, (see supplementary Excel file). After screening and assessment of eligibility, only 12 studies met inclusion criteria—five involving insulin-treated individuals and seven involving non-insulin-treated individuals with T2D with a total of 1206 participants (Figure S1 and Table 1). [20–31]

**Table 1:** Main characteristics of the selected randomized clinical trials

First author	N. of participants	Baseline HbA1c ± SD (%)	Treatment type	Active phase RCT (weeks)	Intermittent use (yes/no)	Type of CGM
Ajjan RA Ref. 20	141	8.6 ± 1.0	Various Insulin therapies	28	Yes	Intermittent scanning
Aronson R Ref. 22	116	8.6 ± 1.1	Non-insulin therapies	16	No	Intermittent scanning
Beck RW Ref. 31	158	8.5 ± 0.6	Multiple daily insulin injections	24	No	Real-time
Bergenstal RM Ref. 21	114	8.0 ± 1.0	Insulin and non-insulin therapies	16	No	Real-time
Cox DJ Ref. 28	30	8.9 ± 1.6	Non-insulin therapies	12	Yes	Real-time
Kim JY Ref. 24	148	8.4 ± 1.0	Multiple daily insulin injections	24	No	Intermittent scanning
Martens T Ref. 27	175	9.1 ± 0.9	Basal insulin + other non-insulin therapies	32	No	Real-time
Moon SJ Ref. 25	61	8.2 ± 0.5	Oral non-insulin therapies	24	Yes	Real-time
Price DA Ref. 26	67	8.4 ± 0.7	Non-insulin therapies	12	Yes	Real-time
Sseemondo E Ref. 23	40	9.6 ± 0.9	Non-insulin therapies	12	No	Intermittent scanning
Wada E Ref. 29	100	7.8 ± 0.3	Non-insulin therapies	24	No	Intermittent scanning
Yaron M Ref. 30	101	8.5 ± 0.8	Multiple daily insulin injections	10	No	Intermittent scanning

We excluded:

- Trials conducted in adolescent populations that were not generalizable to adults with T2DM.
- Trials without a control group.
- Trials in which effects were confounded by additional interventions (e.g., physical activity, weight-loss programs, dietary changes, or prior self-management courses).
- Trials in which insulin-treated and non–insulin-treated participants could not be analyzed separately.

For example, a trial by Allen and colleagues was excluded since the intervention was accompanied by physical activity [32], while the trial reported by Lau et al. was excluded as it also involved remote telemonitoring visits [33]. We also excluded the RCT carried out by Lind et al. due to the fact that all participants received an intensive diabetes self-management education course, which does not correspond to standard clinical practice [34]. The supplementary Excel file details the 937 articles assessed including the specific reasons why they were included or excluded from this metanalysis.

The pharmacoeconomic dimension was also assessed. The included studies employed a range of economic models (predominantly cost utility and cost-effectiveness analyses) and were conducted across multiple countries, including European nations (Scotland, Sweden, Spain, France, the United Kingdom, and Italy), the Americas (the United States, Brazil, and Canada), and East Asia (Japan and China). The payer's perspective was the most frequently adopted.

### Data analysis

The principal outcome of the analysis was the effect of CGM versus SMBG on HbA1c, Time in Range (TIR) and Time Below Range (TBR). Meta-analyses were conducted separately for insulin-treated and non-insulin-treated individuals with type 2 diabetes, according to the predefined PICO questions. Effect sizes were calculated as Standardized Mean Differences (SMD; Hedges’ g) with 95% confidence intervals, allowing combination of studies reporting outcomes on different scales or measurement units. Negative SMDs indicate a reduction in the outcome (e.g., HbA1c, TBR), whereas positive SMDs indicate an increase (e.g., TIR).

Pooled estimates were obtained using a random-effects model, irrespective of the degree of heterogeneity, given the limited number of RCTs available for each comparison. Between-study variance (τ2) was estimated using Restricted Maximum Likelihood (REML), which provides more robust estimates in meta-analyses with limited number of trials.

Statistical heterogeneity was assessed using Cochran’s Q and The I^2^ statistic, interpreted following Cochrane guidance (0–40%: low; 30–60%: moderate; 50–90% substantial; 75–100% considerable), acknowledging that both Q and I^2^ can be unstable when based on few studies.

Potential publication bias was evaluated via visual inspection of funnel plots. No formal tests for asymmetry were performed because the number of available trials per outcome (< 10) precludes reliable small-study effect detection.

All analyses were performed using R (version 4.4.1) and the meta package (version 8.0–2).

The data were analyzed by the panel as standardized means, the results of which are reported in the supplementary Figures (S2 and S3). To improve the clarity for readers, the figures presented in the main text display unstandardized means.

## Results

The main characteristics (number of participants, baseline HbA1c, diabetes treatment and type of CGM) are reported in Table 1.

### Evidence in insulin-treated T2D

**Fig. 1:**
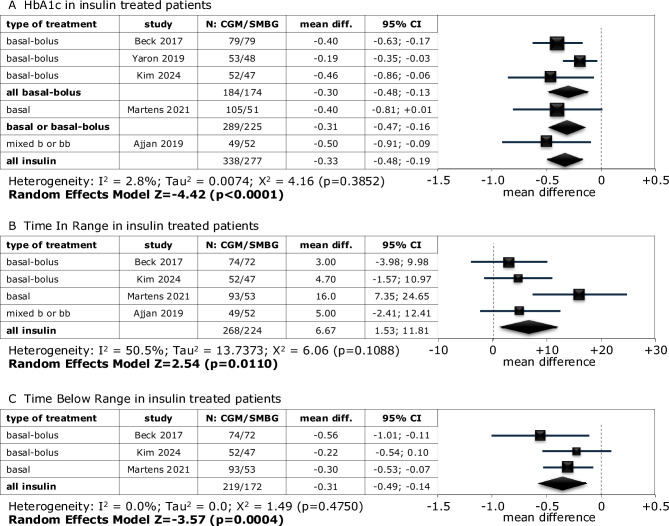
Results of meta-analyses of mean differences of randomized trials comparing the effect of CGM versus SMBG in insulin treated patients. Panel A: HbA1c results. The trials were examined by combining only the studies on patients with diabetes treated with basal-bolus insulin, then a study with basal insulin alone was added, and finally a fifth study conducted on patients treated with both basal-bolus and basal insulin. Panel B: Effect of CGM versus SMBG on time in range. Panel C: Effect of CGM versus SMBG on time below range

The five included RCTs examined CGM in insulin-treated patients and encompassed a mix of basal-bolus and basal-only regimens. Across all studies, CGM consistently demonstrated a statistically significant and clinically meaningful reduction in HbA1c levels. The meta-analysis revealed a standardized mean difference corresponding to an absolute reduction of − 0.33% in HbA1c (95% CI: − 0.48 to − 0.19, p < 0.0001). The consistency of findings across studies, and the low heterogeneity, reinforce the robustness of the result (Fig. 1A). Funnel plots, confirming lack of heterogeneity, are reported in Figure S5.

In insulin-treated patients, use of CGM was also associated with a statistically significant increase in TIR, with an average improvement of + 6.67% (95% CI: + 1.53 to + 11.81, p = 0.0110, Fig. 1B). This was paralleled by a significant reduction in TBR, also averaging -0.31% (95% CI: − 0.49 to − 0.14, p = 0.0004, Fig. 1C). Although blinding was inherently impossible in these studies, overall risk of bias was assessed as low (Figure S4A), consequently the panel graded all the evidence as “moderate” (Figure S5B). Hypoglycemic risk was not directly assessed in the study populations included in the analysis. As these patients were all insulin-treated, we might assume that all studied patients were at risk of hypoglycemia.

### Evidence in non-insulin-treated T2D

**Fig. 2:**
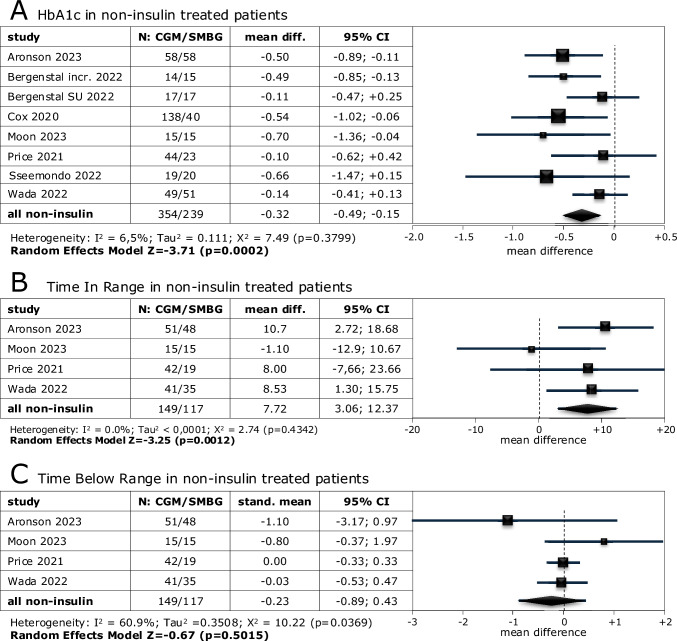
Results of meta-analyses of mean differences of randomized trials comparing the effect of CGM versus SMBG in patients not treated with insulin. Panel A: Effects of CGM versus SMBG on HbA1c. Panel B: Effect of CGM versus SMBG on time in range. Panel C: Effect of CGM versus SMBG on time below range

The meta-analysis once again demonstrated a reduction in HbA1c, this time of -0.32% (95% CI: − 0.49 to − 0.16, p = 0.0002, Fig. 2A) and an increase in TIR of + 7.72% (95% CI: + 3.06 to + 12.37, p = 0.0012, Fig. 2B). However, in contrast with insulin-treated patients, the change in TBR was not statistically significant in this group (Fig. 2C).

Among the seven RCTs conducted in non-insulin-treated patients, the RCT by Bergenstal et al. also permitted subgroup analyses [21], such as comparisons among patients on sulfonylureas or incretin-based therapies (DPP-4 inhibitors and GLP-1 receptor agonists). No significant between-group differences were observed, which may be attributable to the limited sample size.

### Pharmacoeconomic assessment

Economic modeling demonstrated that CGM is a cost-effective intervention for both insulin- and non-insulin-treated individuals, particularly when accounting for potential reductions in acute complications, hospital admissions, and long-term vascular outcomes. Cost-effectiveness was more robustly supported in the insulin-treated group, where more pharmaeconomic studies were available and modeled outcomes were better validated. With the only exception of one study published in Spain in 2018 [35], these findings were broadly consistent across all countries examined (including Italy), regardless of the characteristics of the respective healthcare systems.

### Recommendations

Based on the strength and consistency of the evidence, the panel issued the following recommendations:

A strong recommendation in favor of CGM for individuals with T2D treated with insulin, including those on basal insulin alone.

A strong recommendation was issued in favor of CGM for individuals with T2D not treated with insulin, particularly for those with glycated hemoglobin levels ≥ 7%. The panel selected the HbA1c ≥ 7% threshold based on the lower risk of hypoglycemia in this population, the high mean HbA1c values reported in the reviewed studies, and in alignment with guideline recommended targets, the panel unanimously agreed that the recommendation for CGM use in these patients should mainly apply to those with an HbA1c ≥ 7%. The final document, which includes this analysis and the broader set of national T2D treatment guidelines, was submitted to, revised and approved by the Italian National Institute of Health and is publicly available through the National Guidelines System at https://www.iss.it/-/snlg-terapia-diabete-tipo2

## Discussion

Attaining and maintaining low HbA1c levels remains a core therapeutic target and although the mechanisms of action of diabetes treatments differ, there are consistent indications that reducing HbA1c, particularly to values below 6.5–7.0%, is associated with meaningful reductions in both microvascular and macrovascular events, with up to ~ 27% fewer major cardiovascular events per 1% drop in HbA1c. [35, 36]

The findings from these metanalyses reinforce the clinical value of CGM in reaching target levels of glycated hemoglobin in T2D. Among insulin-treated patients, CGM not only improves glycemic control, but does so to an extent that is almost comparable to the addition of a new glucose-lowering medication. Importantly, these improvements were observed even in those using basal insulin alone, expanding the population that can benefit from CGM beyond those on complex insulin regimens.

Four out of 12 studies [20, 25, 26, 28], utilized CGM intermittently (see Table 1 for details), mostly reporting positive results (significant reduction in primary endpoints). Given the small number of studies, a subgroup analysis was not feasible; however, the available evidence suggests that intermittent CGM use is likely to be effective as well.

The results among non-insulin-treated patients are affirmative. The improvement in HbA1c and TIR across studies suggests that CGM has a role in enhancing self-management and therapeutic adherence, even in the absence of insulin therapy. The lack of side effects, combined with the educational value of CGM, particularly in relation to diet and lifestyle, strengthens the case for its broader implementation.

An interesting observation is that some of the studies used CGM intermittently rather than continuously and yet achieved similar outcomes. This suggests that the primary benefit of CGM may lie not in constant monitoring per se, but in the heightened awareness and behavior modification it fosters. This aligns with the understanding of CGM as a "biofeedback" tool that enhances patients’ understanding of their condition.

Somewhat similar results have been recently described by Ajjan et al. in an expert consensus [20]. The latter, however, included 6 retrospective studies (out of a total of 18 studies), with the longest RCT duration being 52 weeks, and no formal comparison or statistical assessment was performed, thus limiting the strength of the conclusions. A newly published meta-analysis by Aronson et al. also reports similar findings, however, it includes observational studies (which are excluded by Italian rules), which are limited by inherent bias [22]. The authors also differentiated their search on the basis of the various categories of CGM devices. Our meta-analyses avoid the latter differentiation since, given the rapid pace of technological innovation and the risk of obsolescence before long-term data can be published, our panel chose to focus instead on the overall utility of CGM as a concept. This decision avoids the pitfall of making recommendations tied to devices that may no longer be available or supported in the near future.

We excluded trials carried out in adolescent populations and not generalizable to adults with T2DM, and trials lacking control groups; trials in which effects were confounded by additional interventions (e.g., physical activity, weight-loss programs, dietary changes, or prior self-management courses) and trials in which insulin-treated and non–insulin-treated participants could not be analyzed separately. A trial by Allen and colleagues was excluded since the intervention was accompanied by physical activity [32], while the trial reported by Lau et al. was excluded as it also involved remote telemonitoring visits[33] We also excluded the RCT carried out by Lind et al. due to the fact that all participants received an intensive diabetes self-management education course which does not reflect standard clinical practice [34]. The results of the latter study, however, were positive, and its inclusion would have strengthened our findings.

One significant limitation of our evaluation is the decision not to compare individual CGM devices or technologies. Another important limitation of this analysis is the use of the prespecified PICO methodology, requiring the evaluation only of trials in which all enrolled patients were treated with insulin or had no insulin treatment. Several trials had to be excluded from this analysis because not all the patients had similar treatments and data were not described according to the different treatments.

One of the strengths of this update lies in the methodological rigor, transparency, and multidisciplinary nature of the guideline process, which incorporated patient voices at every stage. It is important to note that our results strongly confirm the ADA recommendations 7.15 (use of CGM in patients with type 2 diabetes, whatever the treatment). However, while the ADA recommendations are mostly centered on consensus-based methodology, our results were achieved only after a stringent search of clinical trials and their subsequent meta-analyses, the applicability of which was eventually confirmed after a pharmacoeconomic evaluation. It is also important to note that our Panel was convened and started work before the publication of the 2025 recommendations.

CGM represents a clinically effective and cost-efficient approach to optimizing glycemic control in T2D, particularly among individuals on insulin therapy. Even though our results focused exclusively on glycemic control, it is important to emphasize that use of CGM can reduce acute events and hospitalizations. CGM can improve patient compliance and reduce diabetes-related anxiety while also enhancing empowerment due to the self-management skills it promotes. It can be included in an integrated lifestyle approach to diabetes which includes diet and physical activity. For the healthcare provider, it provides a useful tool to direct treatment options, and rapidly assess the efficacy of new interventions, helping to minimize delays and optimizing HbA1c control. CGM can be used for telemonitoring of patients thus supporting precision medicine.

## Conclusion

In conclusion, our findings support a shift in clinical practice toward the more widespread use of CGM in T2D, with regulatory frameworks and reimbursement policies needing to adapt accordingly. While further long-term studies are warranted on health outcomes and on T2D complications, current evidence strongly supports integrating CGM into the standard of care for T2D.

## Supplementary Information

Below is the link to the electronic supplementary material.

Supplementary file1 (TIFF 1820 KB)

Supplementary file2 (TIFF 1820 KB)

Supplementary file3 (TIFF 1820 KB)

Supplementary file4 (TIFF 1820 KB)

Supplementary file5 (PDF 313 KB)

Supplementary file6 (xlsx 310 KB)
