# Supplementary material for: Evidence-based recommendations for the use of continuous glucose monitoring in type 2 diabetes: the Italian guidelines
Source: Acta Diabetol. 2026 May 28;63(8):1385–94. doi: 10.1007/s00592-026-02693-6 (PMC13469397; doi:10.1007/s00592-026-02693-6)
Supplement: Supplementary file 1 [file 592_2026_2693_MOESM5_ESM.pdf]

PRISMA flow diagram of combined research strategy

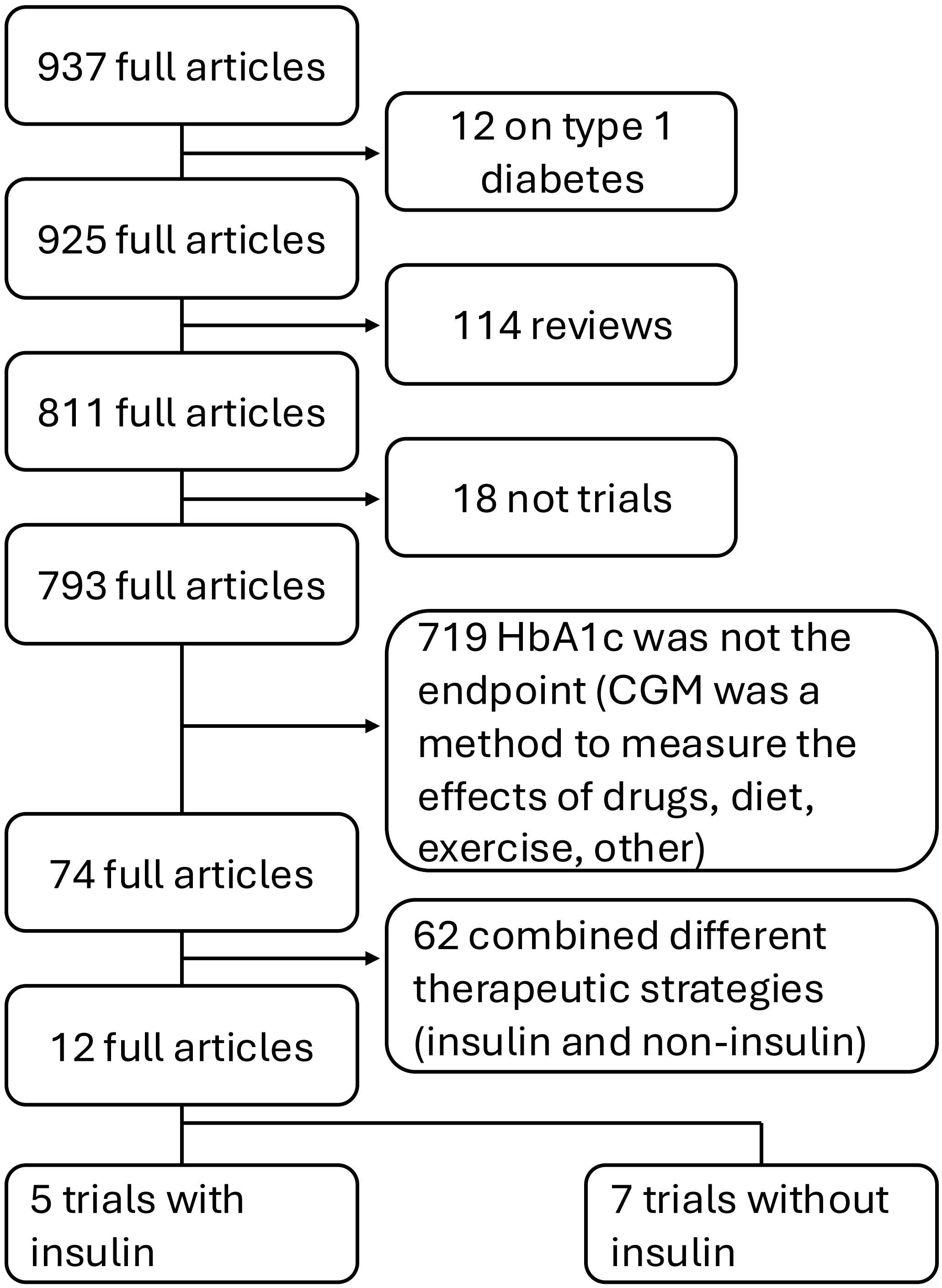

A HbA1c in insulin treated patients

type of treatment	study	N: CGM/SMBG	stand. mean	95% CI
basal-bolus	Beck 2017	79/79	-0.53	-0.85; -0.21
basal-bolus	Yaron 2019	52/47	-0.45	-0.84; -0.05
basal-bolus	Kim 2024	53/48	-0.45	-0.85; -0.05
**all basal-bolus**		184/174	-0.48	-0.69; -0.27
basal	Martens 2021	105/51	-0.35	-0.69; -0.02
**basal or basal-bolus**		289/225	-0.45	-0.63; -0.18
mixed b or bb	Ajjan 2019	52/47	-0.47	-0.86; -0.07
**all insulin**		341/272	-0.45	-0.61; -0.29

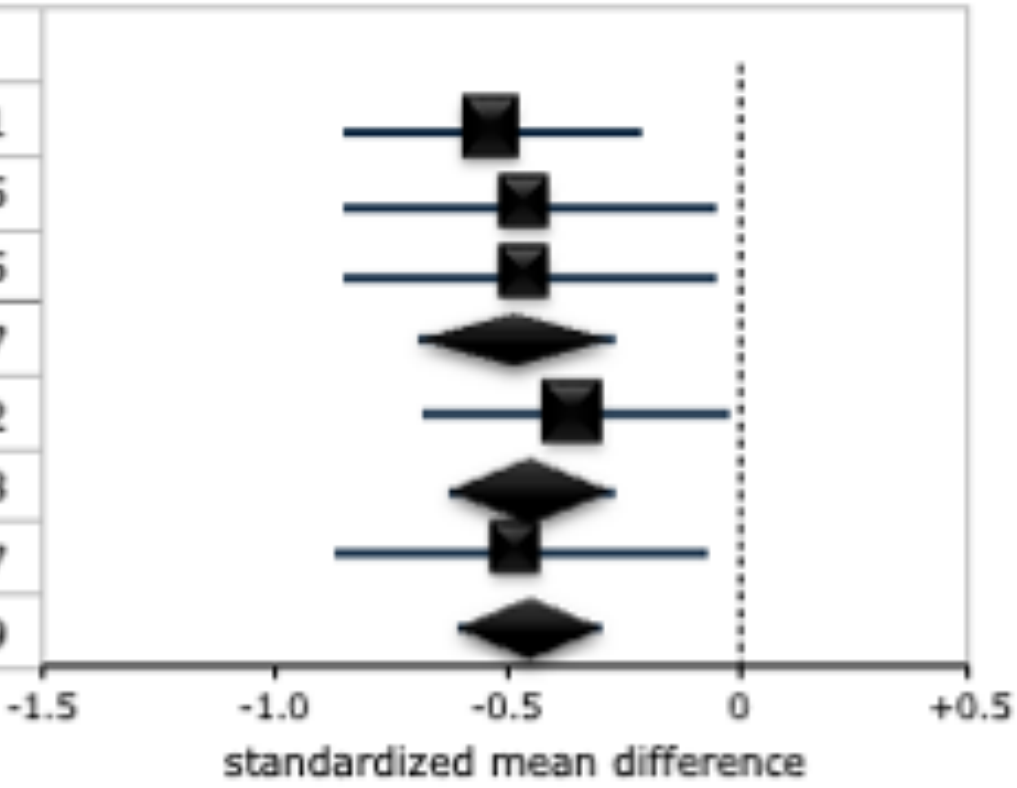

Heterogeneity: $I^2 = 0.0\%$; $Tau^2 = 0$; $X^2 = 0.57$ ($p=0.9669$)
Random Effects Model Z=-5.42 (p<0.0001)

B Time In Range in insulin treated patients

type of treatment	study	N: CGM/SMBG	stand. mean	95% CI
basal-bolus	Beck 2017	74/72	0.14	-0.19; 0.46
basal-bolus	Kim 2024	52/47	0.25	-0.15; 0.65
basal	Martens 2021	93/53	0.63	0.28; 0.97
mixed b or bb	Ajjan 2019	49/52	0.26	-0.13; 0.65
**all insulin**		268/224	0.31	0.10; 0.55

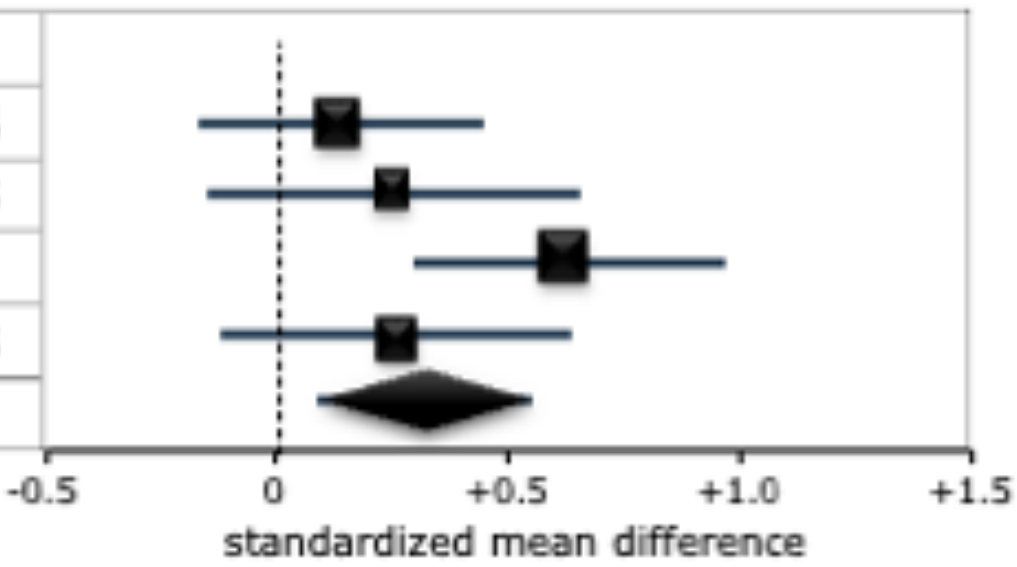

Heterogeneity: $I^2 = 32.7\%$; $Tau^2 = 0.0184$; $X^2 = 4.46$ ($p=0.2163$)
Random Effects Model Z=2.80 (p=0.005)

C Time Below Range in insulin treated patients

type of treatment	study	N: CGM/SMBG	stand. mean	95% CI
basal-bolus	Beck 2017	74/72	-0.40	-0.73; -0.08
basal-bolus	Kim 2024	52/47	-0.28	-0.98; 0.11
basal	Martens 2021	93/53	-0.52	-0.86; -0.17
**all insulin**		219/172	-0.41	-0.61; -0.21

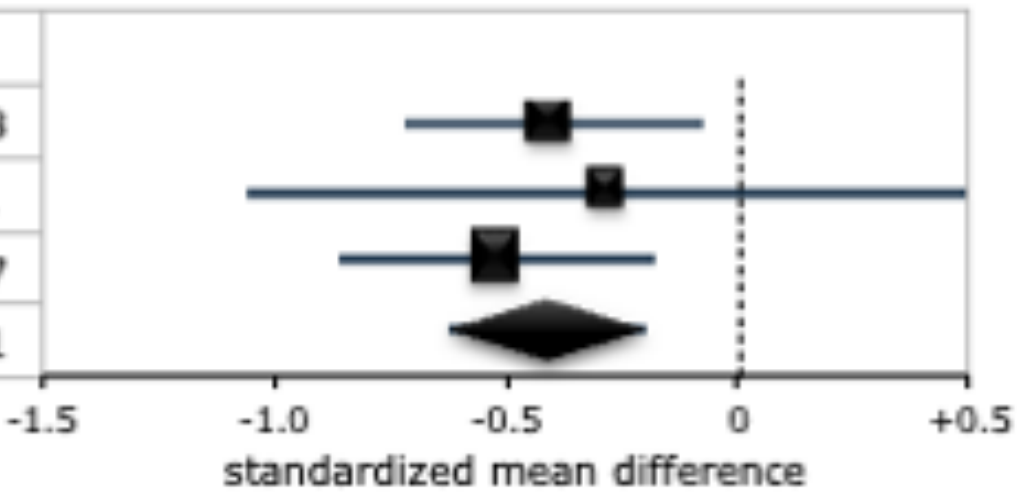

Heterogeneity: $I^2 = 0.0\%$; $Tau^2 = 0.0$; $X^2 = 0.78$ ($p=0.6779$)
Random Effects Model Z=3.96 (p<0.0001)

FIGURE S1.
Results of meta-analyses of randomized trials comparing standardized mean differences of the effect of CGM versus SMBG in insulin treated patients. Panel A: HbA1c results. The trials were examined by combining only the studies on patients with diabetes treated with basal-bolus insulin, then a study with basal insulin alone was added, and finally a fifth study conducted on patients treated with both basal-bolus and basal insulin. Panel B: Effect of CGM versus SMBG on Time In Range. Panel C: Effect of CGM versus SMBG on Time Below Range.
Risk of bias and GRADE evaluations are in Figure S4; funnel plots are in figure S5.

A HbA1c in insulin treated patients

type of treatment	study	N: CGM/SMBG	stand. mean	95% CI
basal-bolus	Beck 2017	79/79	-0.53	-0.85; -0.21
basal-bolus	Yaron 2019	52/47	-0.45	-0.84; -0.05
basal-bolus	Kim 2024	53/48	-0.45	-0.85; -0.05
**all basal-bolus**		184/174	-0.48	-0.69; -0.27
basal	Martens 2021	105/51	-0.35	-0.69; -0.02
**basal or basal-bolus**		289/225	-0.45	-0.63; -0.18
mixed b or bb	Ajjan 2019	52/47	-0.47	-0.86; -0.07
**all insulin**		341/272	-0.45	-0.61; -0.29

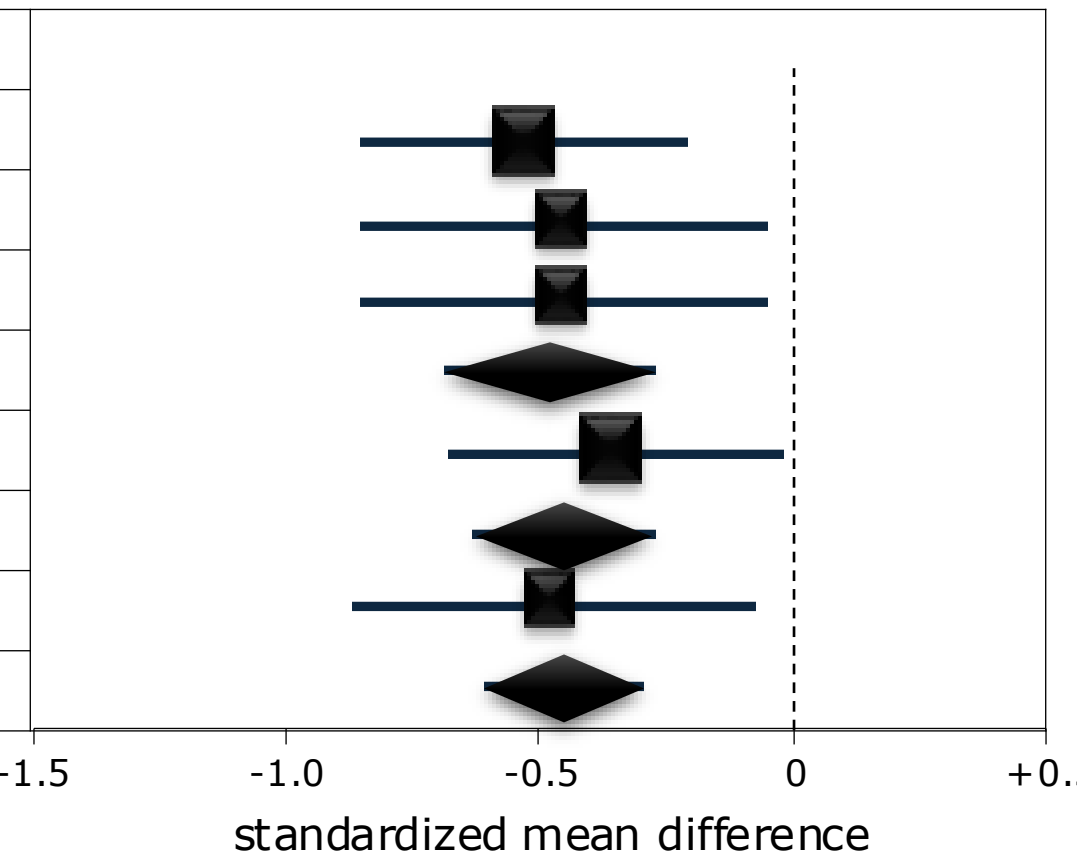

Heterogeneity: $I^2 = 0.0\%$; $Tau^2 = 0$; $X^2 = 0.57$ ($p=0.9669$)
Random Effects Model Z=-5.42 (p<0.0001)

B Time In Range in insulin treated patients

type of treatment	study	N: CGM/SMBG	stand. mean	95% CI
basal-bolus	Beck 2017	74/72	0.14	-0.19; 0.46
basal-bolus	Kim 2024	52/47	0.25	-0.15; 0.65
basal	Martens 2021	93/53	0.63	0.28; 0.97
mixed b or bb	Ajjan 2019	49/52	0.26	-0.13; 0.65
**all insulin**		268/224	0.31	0.10; 0.55

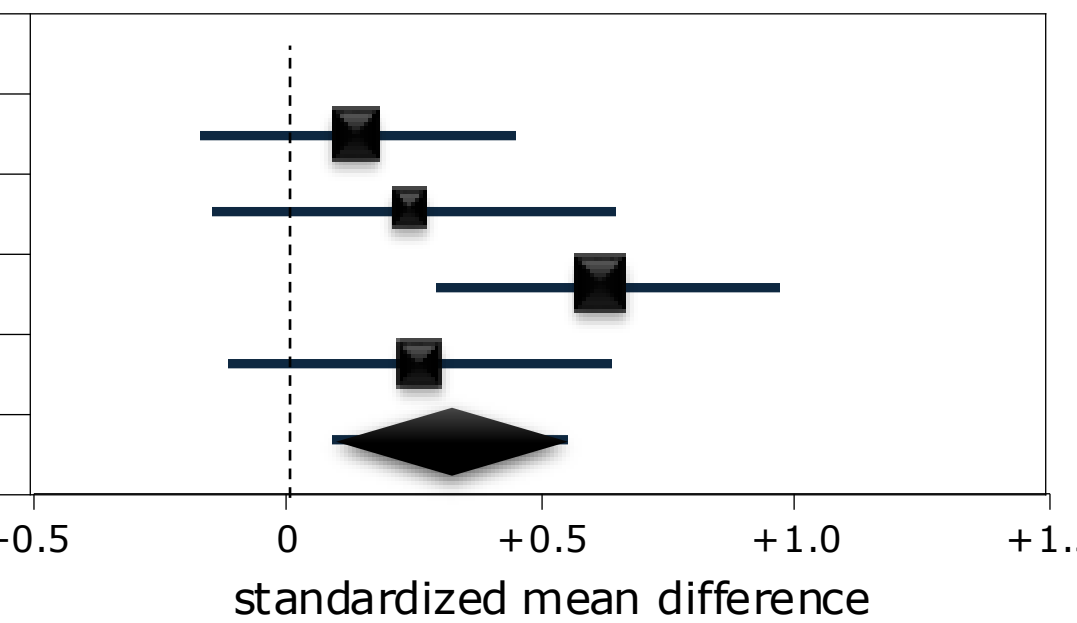

Heterogeneity: $I^2 = 32.7\%$; $Tau^2 = 0.0184$; $X^2 = 4.46$ ($p=0.2163$)
Random Effects Model Z=2.80 (p=0.005)

C Time Below Range in insulin treated patients

type of treatment	study	N: CGM/SMBG	stand. mean	95% CI
basal-bolus	Beck 2017	74/72	-0.40	-0.73; -0.08
basal-bolus	Kim 2024	52/47	-0.28	-0.98; 0.11
basal	Martens 2021	93/53	-0.52	-0.86; -0.17
**all insulin**		219/172	-0.41	-0.61; -0.21

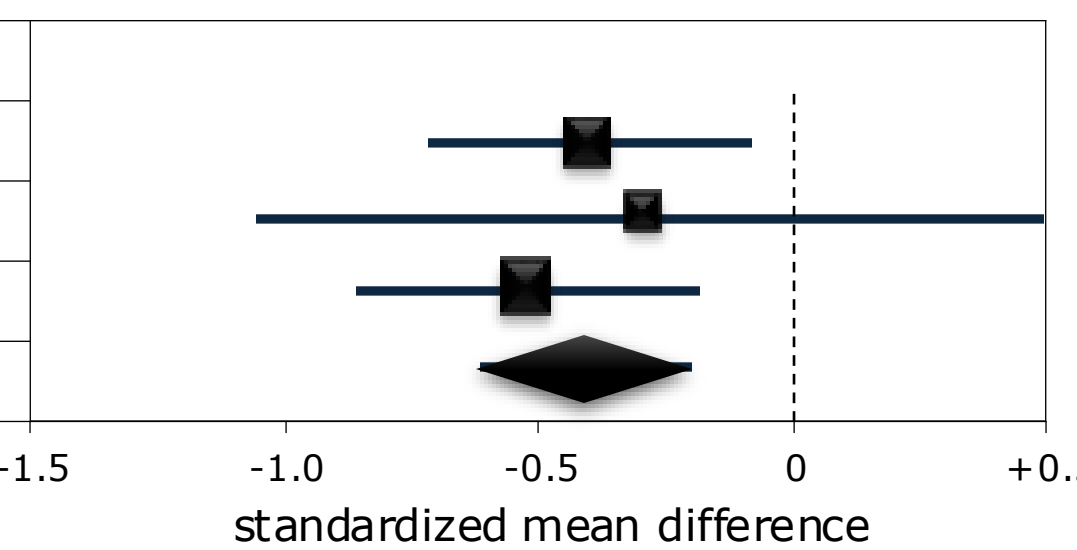

Heterogeneity: $I^2 = 0.0\%$; $Tau^2 = 0.0$; $X^2 = 0.78$ ($p=0.6779$)
Random Effects Model Z=3.96 (p<0.0001)

FIGURE S2.
Results of meta-analyses of randomized trials comparing standardized mean differences of the effect of CGM versus SMBG in insulin treated patients. Panel A: HbA1c results. The trials were examined by combining only the studies on patients with diabetes treated with basal-bolus insulin, then a study with basal insulin alone was added, and finally a fifth study conducted on patients treated with both basal-bolus and basal insulin. Panel B: Effect of CGM versus SMBG on Time In Range. Panel C: Effect of CGM versus SMBG on Time Below Range.
Risk of bias and GRADE evaluations are in Figure S4; funnel plots are in figure S5.

A HbA1c in non-insulin treated patients

study	N: CGM/SMBG	stand. mean	95% CI
Aronson 2023	58/58	-0.47	-0.84; -0.10
Bergenstal incr. 2022	14/15	-0.95	-1.72; -0.17
Bergenstal SU 2022	17/17	-0.20	-0.88; 0.47
Cox 2020	138/40	-0.40	-0.76; -0.05
Moon 2023	15/15	-0.74	-1.48; -0.00
Price 2021	44/23	-0.09	-0.60; 0.41
Wada 2022	49/51	-0.20	-0.59; 0.20
Sseemondo 2022	19/20	-0.19	-0.82; 0.44
**all non-insulin**	354/239	-0.36	-0.53; -0.19

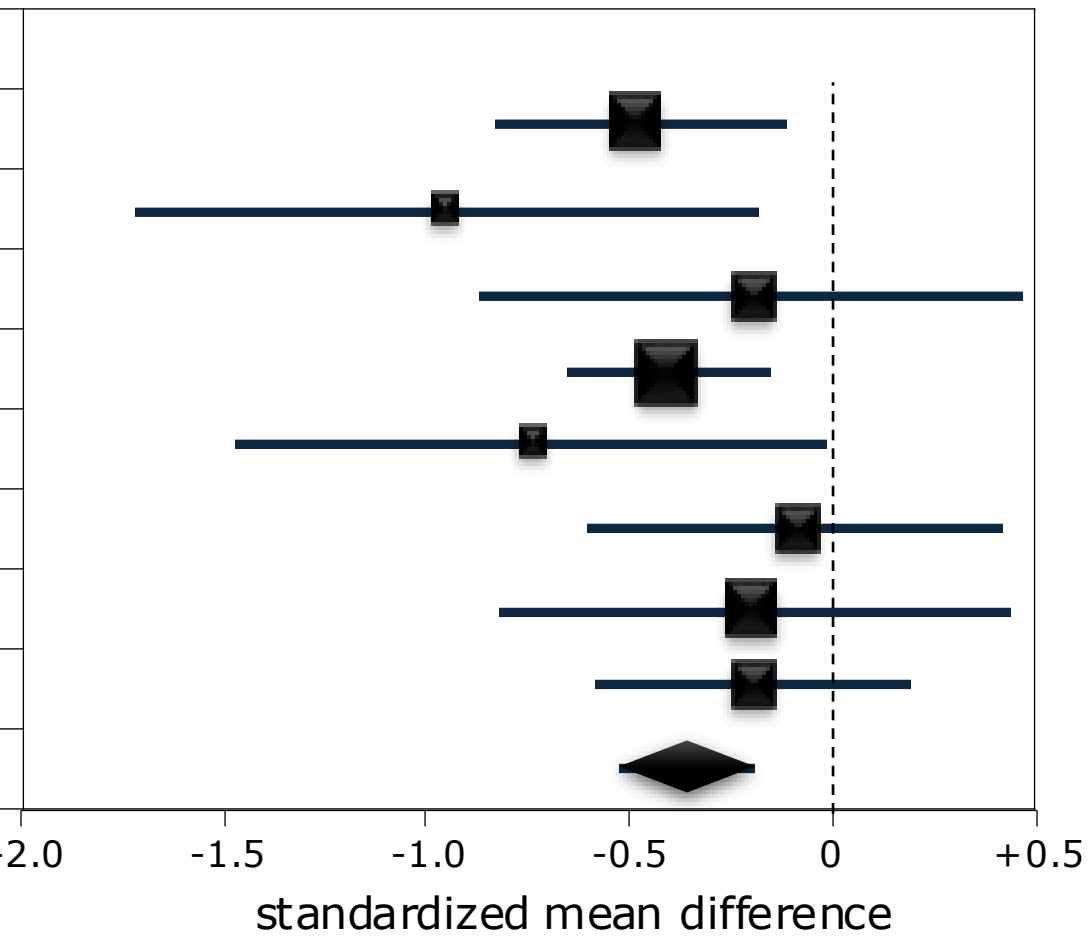

Heterogeneity: I^2 = 0.0%; Tau^2 = 0; X^2 = 5.85 (p=0.5570)
Random Effects Model Z=-4.09 (p=0.000)

B Time In Range in non-insulin treated patients

study	N: CGM/SMBG	stand. mean	95% CI
Aronson 2023	51/48	0.53	0.13; 0.93
Moon 2023	15/15	-0.06	-0.78; -0.65
Price 2021	42/19	0.29	-0.25; 0.84
Wada 2022	41/35	0.54	0.08; 1.00
**all non-insulin**	149/117	0.41	0.16; 0.66

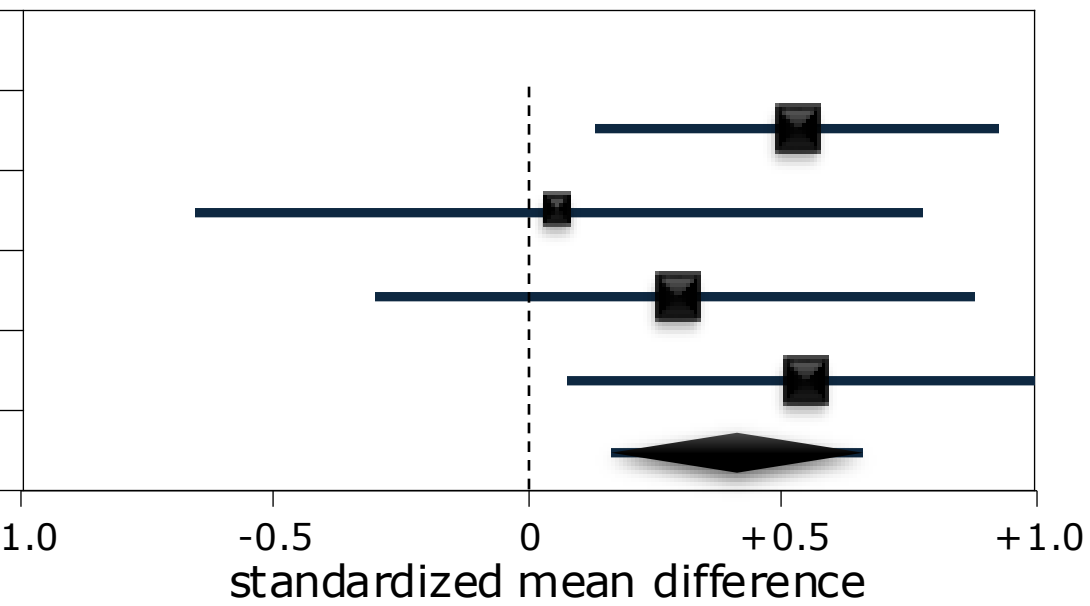

Heterogeneity: I^2 = 0.0%; Tau^2 < 0,0001; X^2 = 2.52 (p=0.4723)
Random Effects Model Z=-3.26 (p<0.0001)

C Time Below Range in non-insulin treated patients

study	N: CGM/SMBG	stand. mean	95% CI
Aronson 2023	51/48	-0.21	-0.61; 0.18
Moon 2023	15/15	0.48	-0.25; 1.20
Price 2021	42/19	0.00	-0.54; 0.54
Wada 2022	41/35	-0.03	-0.48; 0.42
**all non-insulin**	149/117	-0.03	-0.28; 0.21

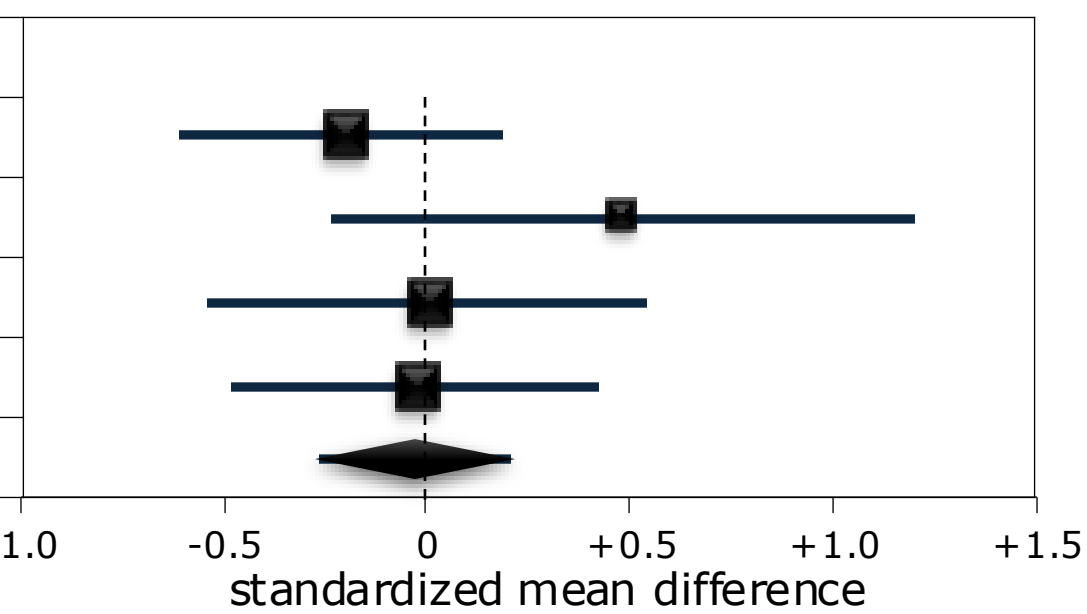

Heterogeneity: I^2 = 0.0%; Tau^2 < 0,0001; X^2 = 2.68 (p=0.4435)
Random Effects Model Z=-0.28 (p=0.780)

FIGURE S3.
Results of meta-analyses of randomized trials comparing standardized mean differences of the effect of CGM versus SMBG in patients not treated with insulin. Panel A: Effects of CGM versus SMBG on HbA1c. Panel B: Effect of CGM versus SMBG on Time In Range. Panel C: Effect of CGM versus SMBG on Time Below Range. Risk of bias and GRADE evaluations are in Figure S4; funnel plots are in figure S5.

A
Risk of bias

A Random sequence generation (selection bias)

B Allocation concealment (selection bias)

C Blinding of participants and personnel (performance bias)

D Blinding of outcome assessment (detection bias)

E Incomplete outcome data (attrition bias)

F Selective reporting (reporting bias)

G Other bias

	A	B	C	D	E	F	G
INSULIN							
Beck	+	+	–	–	+	+	+
Ajjan	+	+	–	–	+	–	+
Martens	+	+	–	–	+	+	+
Yaron	+	+	–	–	+	+	–
Kim	+	+	–	–	+	–	+
NO INSULIN							
Moon	+	+	–	–	+	–	+
Aronson	+	+	–	–	+	+	+
Bergenstal	+	+	–	–	+	+	+
Wada	+	+	–	–	+	+	+
Price	+	+	–	–	+	+	+
Cox	+	+	–	–	+	+	–
Sseemondo	+	+	–	–	+	+	+

B
GRADE evaluation

Type of studies	Number of studies	Risk of bias	Inconsistency of results	Lack of generalizability	Imprecision	Other considerations	Grade	Standard. Mean Difference	95% CI	Quality	Importance
INSULIN HbA1c											
RCT	5	minimal	no	minimal	minimal	consistent results	+++ moderate	-0.45	-0.61: -0.29	moderate	moderate
INSULIN TIR											
RCT	4	minimal	no	minimal	Minimal	consistent results	+++ moderate	0.31	0.10; 0.55	moderate	moderate
INSULIN TBR											
RCT	3	minimal	no	minimal	minimal	consistent results	+++ moderate	-0.41	-0.61; -0.21	moderate	moderate

NO INSULIN HbA1c											
RCT	7	minimal	minimal	minimal	minimal	significant	+++ moderate	-0.36	-0.53; -0.19	moderate	moderate
NO INSULIN TIR											
RCT	4	minimal	minimal	minimal	minimal	significant	+++ moderate	0.41	0.16; 0.66	moderate	moderate
NO INSULIN TBR											
RCT	4	minimal	high	some	yes	not significant	+ very low	-0.03	-0.28; 0.21	moderate	moderate

FIGURE S4.
Panel A: Risk of bias. Panel B: Summary of the GRADE evaluation.

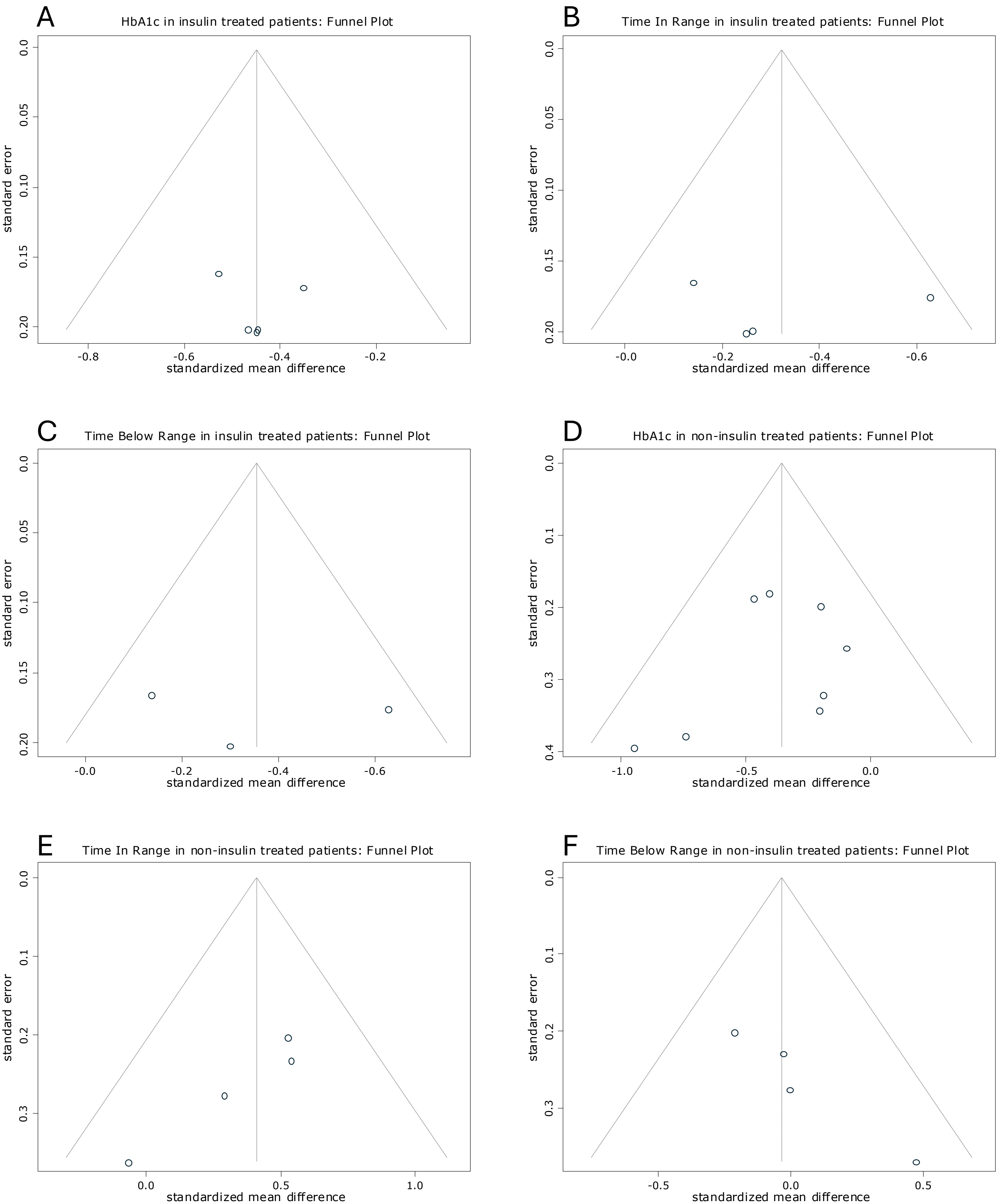

FIGURE S5.
Funnel plots for studies in insulin treated patients (A: HbA1c; B: Time in Range; C: Time below range) and studies in non-insulin treated patients (D: HbA1c; E: Time in Range; F: Time below range)
